# Barriers and facilitators to the implementation of a structured visual assessment after stroke in municipal health care services

**DOI:** 10.1186/s12913-021-06467-4

**Published:** 2021-05-24

**Authors:** Torgeir S. Mathisen, Grethe Eilertsen, Heidi Ormstad, Helle K. Falkenberg

**Affiliations:** 1 National Centre for Optics, Vision and Eye Care, Faculty of Health and Social Sciences, University of South-Eastern Norway, Hasbergs vei 36, 3616 Kongsberg, Norway; 2 USN Research Group of Older Peoples’ Health, University of South-Eastern Norway, Drammen, Norway; 3 Department of Nursing and Health Science, Faculty of Health and Social Sciences, University of South-Eastern Norway, Drammen, Norway; 4 University of South-Eastern Norway, Drammen, Norway

**Keywords:** Stroke, Vision, Visual impairments, Knowledge translation, Implementation, Barriers, Rehabilitation

## Abstract

**Background:**

Stroke is a leading cause of disability worldwide. Visual impairments (VIs) affect 60% of stroke survivors, and have negative consequences for rehabilitation and post-stroke life. VIs after stroke are often overlooked and undertreated due to lack of structured routines for visual care after stroke. This study aims to identify and assess barriers and facilitators to the implementation of structured visual assessment after stroke in municipal health care services. The study is part of a larger knowledge translation project.

**Methods:**

Eleven leaders and municipal interdisciplinary health care professionals participated in qualitative interviews. During two workshops, results from the interviews were discussed with 26 participants from municipal health care services and user representatives. Data from interviews and workshops were collected before the intervention was implemented and analyzed using content analysis.

**Results:**

The analysis identified individual and contextual barriers and facilitators. The individual barriers were related to the participants' experiences of having low competence of visual functions and vision assessment skills. They considered themselves as generalists, not stroke experts, and some were reluctant of change because of previous experiences of unsuccessful implementation projects. Individual facilitators were strong beliefs that including vision in stroke care would improve health care services. If experienced as useful and evidence based, the new vision routine would implement easier. Contextual barriers were experiences of unclear responsibility for vision care, lack of structured interdisciplinary collaboration and lack of formal stroke routines. Time constraints and practical difficulties with including the vision tool in current medical records were also expressed barriers. Contextual facilitators were leader support and acknowledgement, in addition to having a flexible work schedule.

**Conclusions:**

This study shows that improving competence about VIs after stroke and skills in assessing visual functions are particularly important to consider when planning implementation of new vision routines in municipal health care services. Increased knowledge about the consequences of living with VIs after stroke, and the motivation to provide best possible care, were individual facilitators for changing clinical practice. Involving knowledge users, solutions for integrating new knowledge in existing routines, along with easily accessible supervision in own practise, are essential facilitators for promoting a successful implementation.

**Supplementary Information:**

The online version contains supplementary material available at 10.1186/s12913-021-06467-4.

## Background

Knowledge translation (KT) is a systematic process with the aim to bridge the gap between knowledge and practise. KT includes the identification and synthesis of evidence and an active strategy to implement the evidence in a specific practice [1]. The knowledge to action model (KTA) by Graham and colleagues [2] describes the important phases in the KT process. It is a process model frequently used in clinical health care settings [3] and has two main components: 1) knowledge creation (knowledge inquiry, synthesis and tools/procedures) and 2) action cycle (adapt knowledge to the local context, identify barriers, tailor and implement interventions, monitor, evaluate and sustain knowledge). A crucial element that influence the outcome of the implementation is identifying and addressing barriers and facilitators for knowledge use [1], which is the focus of the current study.

Worldwide, stroke is a leading cause of death and disability [4]. One of many sequelae after stroke is visual impairments (VIs), which can affect over 60% of all stroke survivors [5]. VIs after stroke include visual field defects, eye movement disorders, reduced visual acuity and perceptual disorders [5, 6]. VIs after stroke are associated with an increase in depression, falling, decreased participation in activities and a reduced effect of general rehabilitation, among others [7–9].

The symptoms of VIs before and after stroke can be difficult to identify and be misinterpreted as other problems [10–15]. Vision-related symptoms such as dizziness, reading problems, headache, balance problems, and fatigue are not always experienced as a visual problem by the stroke survivor [10, 11]. For example, people with visual field defects after stroke may lack a conscious awareness that large parts of their visual field are missing or that the brain is filling in the empty space with something sensible [12, 13]. This can complicate their understanding of their visual problem [16] and may lead to underreporting symptoms of VIs from the stroke survivors themselves. Unless the visual function is properly examined, many visual symptoms are difficult to identify by health care professionals (HCP) and may be overlooked or perceived as a symptom of other impairments [14]. To secure proper care and rehabilitation, it is crucial that visual function is assessed in health services.

In Norway and internationally, there are lack of national care pathways for VIs after stroke in health care services [15–18], which has led to a variation in the quality of assessment and follow-up of VIs in stroke care [19]. In stroke services, there is a gap between research evidence on how to assess and follow up on VIs after stroke and clinical practice [11, 20]. Stroke survivors experience little or no follow-up and rehabilitation of VIs after discharge from acute stroke care [13, 21, 22]. Therefore, a strengthening of vision competence in stroke care to identify VIs and initiate early rehabilitation is needed [23, 24]. In Norway, the hospital stay in stroke units is short (median 5 days) [25], and municipal health care services are the main providers of primary care, including rehabilitation and follow-up after the initial treatment [25–27]. A recent article from our group confirmed that Norwegian stroke survivors experienced a lack of attention and follow-up of VIs after stroke and that HCP in both specialist and municipal health care services had their focus and competence on the other consequences of stroke [13]. This necessitates the need for vision competence and attention in municipal stroke services because of municipal health care services important role in stroke care and rehabilitation.

In stroke services, several functional assessment tools are implemented, but there is no standard tool that includes a full vision assessment [28]. The Vision Impairment Screening Assessment (VISA) tool has been validated in the UK; with this tool, clinicians who are not specialists in vision problems can identify VIs and refer patients with VIs to vison experts [29]. A similar assessment tool, Competence, Rehabilitation of Sight after Stroke (KROSS), was developed and implemented in two Norwegian stroke units [30]. Both tools assess visual acuity, eye alignment and movements, visual field and visual inattention [29, 30]. In addition, the patients are asked about symptoms, and clinical observations are described. It was designed to provide a non-vision expert HCP with an easy-to-use tool to help identify VIs after stroke during treatment in the stroke unit. KROSS was introduced to two stroke units during a two-day vision-after-stroke workshop with theoretical and practical education, and it has been a useful tool in these settings [30]. The current study aimed to identify the barriers and facilitators of importance to implement the KROSS visual assessment tool after stroke in municipal health care services.

## Methods

**Fig. 1:**
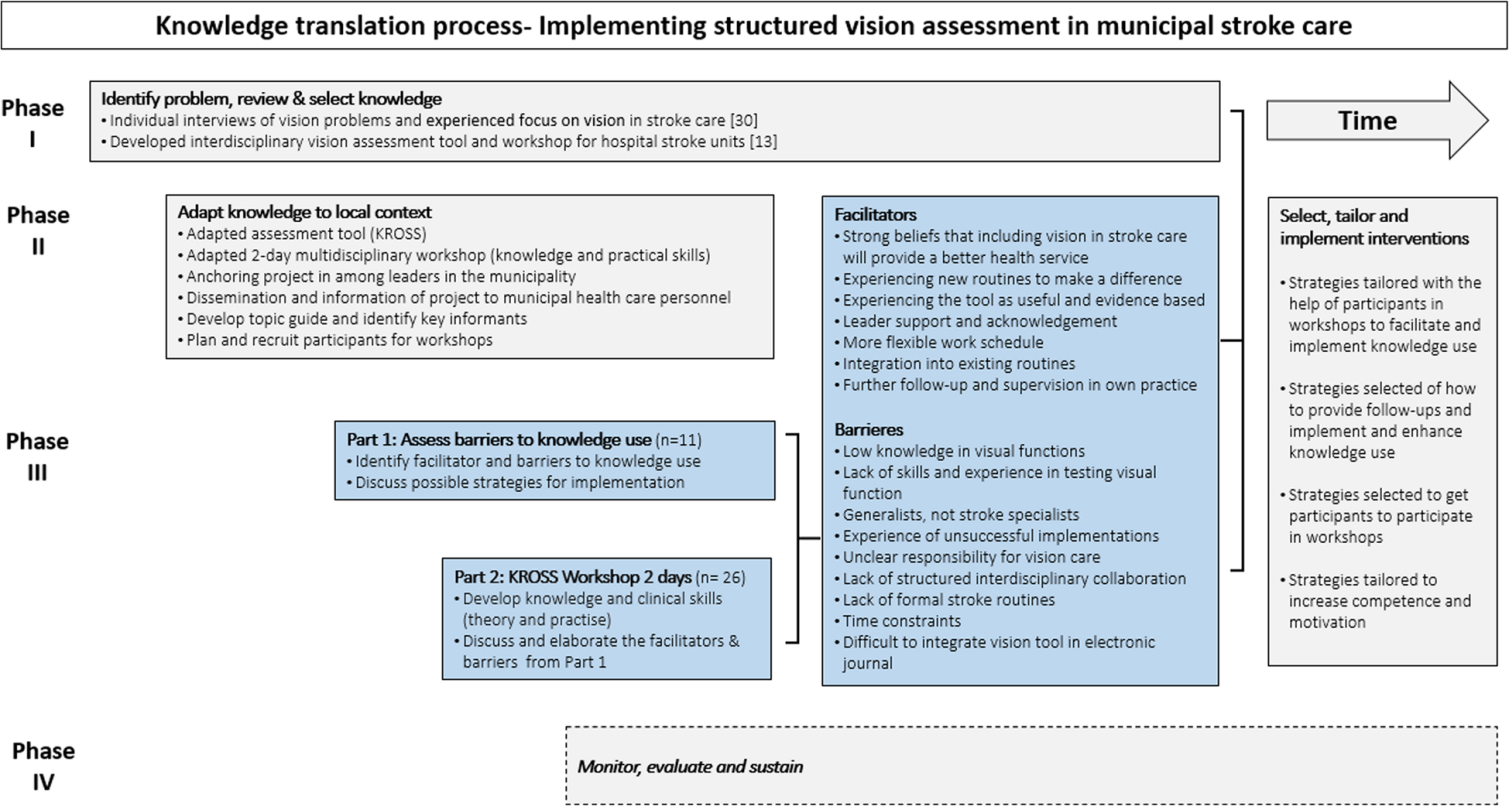
An overview of the four KT phases in this implementation project. Phase III (blue boxes) is the focus of the current study and describes how the interviews and workshops were used to assess barriers and facilitators. Phase I has been described elsewhere [13, 30]. Phase II describes the process of how knowledge was adapted to the local context in preparation for phase III. Phase IV will be the content of a later publication

This qualitative study describes phase III of a larger KT project, where the overall aim is to implement structured vision assessment and follow-up of VIs in municipal health care services after stroke (see Fig. 1). A qualitative approach was chosen because there is limited knowledge of the determinants of implementing knowledge of VIs after stroke in municipal health care services [31]. We considered it important to secure in-depth and broad descriptions of potential barriers and facilitators. The material consists of qualitative individual interviews with 11 HCP and group discussions with 26 HCP participants from two different KROSS workshops.

### Setting and partner involvement

This study took place in a medium-sized Norwegian municipality. In Norway, the health care services are mainly publicly funded [27], and the municipals are responsible for providing primary health care services including general practitioner, prevention, treatment, rehabilitation and palliative care [27]. The most commonly used municipal health services for stroke survivors are the in-patient rehabilitation unit, home-based rehabilitation and home care [25] which are the three services involved in this KT project. Figure 1 describes details of the KT phases I-IV in this implementation project, including that the phases start at different times but overlap. This study focuses on assessing barriers and facilitators to implementing vision assessment after stroke (Phase III, Fig. 1).

The municipality, the Norwegian Association of the Blind and Partially Sighted, the Norwegian Association for Stroke Survivors and the Norwegian Heart and Lung Foundation (LHL Hjerneslag) were active partners in the planning and execution of this project to secure a participative approach. The research group had several meetings with municipal leader groups to inform, adapt, anchor, and engage leaders and service managers in the project’s implementation. Initially, with the head of municipal health care services and service leaders, subsequently with managers in the in-patient rehabilitation, home rehabilitation, home care and the service allocation office (Phase II, Fig. 1). In these meetings, the background for the project and the possible barriers to implementation of visual assessment after stroke were discussed. Together, suggestions on practical considerations and key persons to involve from the different services were provided. Both leaders and service managers confirmed there was a lack of procedures and attention toward VIs after stroke in municipal health care services, and they recognised the value and need for improvement. To enhance the relevance and promote success of this implementation, knowledge users (municipal nurses, nurses assistants, physiotherapists, and occupational therapists) were also active partners, in line with integrated knowledge translation (IKT) [4, 5]. Knowledge users and stroke survivors were involved during the planning and preparation of the interviews, implementation and workshops [32, 33]. This active partner involvement gave common understanding of the project’s aim and the importance of improving services for stroke survivors with VIs (Phase II, Fig. 1.)

### Data collection

#### Participants and recruitment

The participants were purposefully recruited for the individual interviews to secure representation of relevant health care professions in the three municipal health services. Service managers informed and invited HCP they believed had valuable insights to share with the research group. All HCP consenting to participate were included in the study. The final sample comprised 11 health care professionals: six nurses, four physiotherapists and one occupational therapist. Invitations to the KROSS workshops were sent out from the head of municipal health care services to all employees in the rehabilitation unit, the home rehabilitation service and home care services. The service managers facilitated and encouraged their staff to participate. Twenty-two interdisciplinary health care professionals participated, representing the three municipal services, service managers and staff from the service allocation office. In addition, three participants from a specialist rehabilitation hospital and one from an acute stroke unit participated in the workshops on their own requests after learning about the project from one of the user groups. The 26 participants were nurses, physiotherapists, occupational therapists and assistant nurses. Three participants from the workshop also participated in the individual interviews. Four stroke survivors with VIs participated in both the theoretical and practical parts of the workshops. All had visual field loss, in combination with at least one other VIs, including reduced visual acuity and/or ocular motility problems. Their experiences living with VIs ranged from 3 months to several years. Two represented the patient organisations and were actively involved in planning the project, and two were recruited from the municipal services.

#### Interviews

The interviews were semi-structured using a topic guide to ensure key areas were covered. The topics were based on input from all partners, patient experiences [13] and determinants frequently reported in the literature [34–38]. In addition to specific questions about their knowledge of, attention to and practice on VIs, we also asked general questions about experiences related to the implementation of new assessment tools, leader involvement and how they viewed the climate for competence improvement. The topic guide is available as a supplementary material S1. The interviews took place at the university, or in a neutral location chosen by the participants. The participants were encouraged to freely describe their views and experiences on the assessment and follow-up of VIs after stroke.

Because of practical reasons, three participants working in home care were interviewed as a group. All interviews were completed before the workshop, and preliminary results were used to adapt the workshop to the local context [2]. The interviews lasted from 30 to 75 min. Except for one individual interview, in which notes were taken during the interview because of the participant’s preferences, the interviews were recorded and transcribed verbatim.

#### KROSS workshop

The KROSS workshop was part of the implementation strategy in this KT project. The KROSS workshop was designed earlier as part of developing the KROSS tool for use in two hospital stroke units [30]. The workshop was adapted to the local municipal setting in collaboration with all partners and preliminary results from the individual interviews (Phase II, Fig. 1). The KROSS workshops were provided by the two first authors (a nurse and an optometrist) in the university’s clinic over 2 days one week apart (Supplementary material S2). The workshop was repeated twice. The content of the workshop consisted of theoretical education on vision and stroke, assessment of VIs, and practical training using the KROSS tool. The stroke survivors contributed with their experiences of living with VIs after stroke, participated in the discussions and acted as demonstration patients during practical training. The workshop included a reflection discussion of barriers and facilitators identified in the interviews to enable a wider group of HCP to elucidate and provide practical insights on how to promote a successful implementation, now that they had more knowledge of VIs and stroke, and had practiced using the KROSS tool. Notes were taken during these discussions.

#### Data analysis

The interviews and notes from the workshop reflections were analysed using an inductive content analysis as described by Graneheim and Lundmann [39, 40]. NVivo 12 was used to manage the data during the analysis. The entire interview text and notes from the workshops were read as a whole several times by TSM and HKF. TSM started to identify the meaning units by marking a part of the text that represented an expressed meaning related to the area of interest. Meaning units were condensed to a shorter form while still preserving its content before being grouped together with other meaning units with similar content into subcategories. TSM and HKF discussed the subcategories and their connection to each other, and all authors discussed and agreed on the subcategories and categories. The levels of interpretation of the subcategories and categories were kept close to the text (manifest content), in line with Graneheim and Lundmann [39]. Through the analysis it became clear that the different barriers and facilitators were related to the individual professional or their professional context.

## Results

The analysis showed individual and contextual barriers and facilitators of importance when it comes to implementing a structured visual assessment after stroke in municipal health care service (see Table 1). Tables 2, 3, 4, 5 describe each barrier and facilitator with quotes.

**Table 1:** The results presented as the participants’ experiences of individual and contextual barriers and facilitators

Participants’ experiences of individual and contextual facilitators and barriers	
Individual	Contextual
**Barriers** i. Low knowledge about visual functions ii. Lack of skills and experience in testing visual function iii. Generalists, not stroke specialists iv. Experience of unsuccessful implementationsi.	**Barriers** i. Unclear responsibility for vision care ii. Lack of structured interdisciplinary collaboration iii. Lack of formal stroke routines iv. Time constraints v. Difficult to integrate vision tool in the medical record
**Facilitators**	**Facilitators**
i. Strong beliefs that including vision in stroke care would provide a better health service ii. Experiencing new routines to make a difference iii. Experiencing the tool as useful and evidence based	i. Leader support and acknowledgement ii. More flexible work schedule iii. Integration into existing routines iv. Further follow-up and supervision in own practice

**Table 2:** Individual barriers illustrated with quotes

Individual barriers	Quotes
Low competence about visual functions	*Several of us have now discussed that we really have not been thinking much about it [VIs after stroke], other than neglect of course. I can’t really remember that I really learned much more about it in school either (P10).*
Lack of skills and experience in testing visual function	*I try to test eye movements. If they can see in all directions, eh and visual field defects but I don’t feel confident and qualified that I am doing it right. However, I do get an impression if you know what I mean, but I am not sure if it is exactly right. But I get an impression if it may be something with the vision (P5).*
Generalists, not stroke specialists	*We have so many groups of patients, from functional decline, hip fractures, COPD and a lot of Parkinson lately. So, it varies how many stroke patients we have (P6).*
Experience of unsuccessful implementation	*It [implementation of new routines] often works fine the first week, and suddenly it is put a side. I don’t think it is bad* ***will****, because everyone agrees. It is easy to fall back to old habits, and suddenly it seems like it is forgotten in a way. Yes, it takes time to make a change. We keep going back to the old routines (P8).*

**Table 3:** Individual facilitators illustrated with quotes from the participants

Individual facilitators	Quotes
Strong beliefs that including vision in stroke care will provide a better health service	*It needs to be implemented because it is important for the patients. When you know the large number of stroke survivors with VIs and when many do not discover it themselves its reason enough for us to be systematic in the assessment of it. It’s about contributing to better lives (P8).* *I experience stroke patients as very motivated, in a way, to get better. Because of the acute changes to their functional level, it triggers something in many, and they want to get back to where they were. Therefore, it is important to be prepared and be able to receive them and provide a good assessment (P6).*
Experiencing new routines to make a difference	*I believe it is important that we experience it [vision routine] as useful. That we can use it immediately. In rehabilitation, and of course, for further recommendations and referrals (P2).*
Experiencing the tool as useful and evidence based	*It is important for me when I am going to use the test results to show something or to show a change, that it [vision assessment tool] is standardised and validated (P9).* *I think it should be relevant. That it serves a purpose, that it’s not just a formality but is useful and has a meaning. The other things are not that important to me, I am not a stickler (P2).*

**Table 4:** Contextual barriers illustrated with quotes from the participants

Contextual barriers	Quotes
Lack of formal stroke routines	*In our municipality there is no formal procedure for a stroke pathway when the patient is transferred from the hospital to municipal health care service … (),.. our assessments are not systematic; they are random and depend on each professional (P1).*
Unclear responsibility for vision care	*The responsibility for follow-up of vision is fragmented. Like in the rehabilitation ward, everyone should be aware of VIs, but maybe some should be specialists in assessing it (P1).*
Lack of structured interdisciplinary collaboration	*You do not need to be a physiotherapist to perform or explain different tests. But, often it becomes the physiotherapist’s job to perform it in practice because we have time to get involved with the patients. So, eh it often ends up to be a task for the physiotherapists alone (P7).*
Time constraints	*It is important that it does not take a long time to perform. And that it’s not too complicated, while still giving us information if something is wrong and we need to refer for follow-up (P5).*
Difficult to integrate vision tool in the medical record	*We have I pads with us, but assessments tools can’t be used directly on the I pads. That is something we want, so we can register in the results while we perform the test in the patients home. (P3).*

**Table 5:** Contextual facilitators illustrated with quotes from the participants

Contextual facilitators	Quotes
Leader support and acknowledgement	*After working for some years, I think the need for leader involvement varies from person to person. I see that some need more follow-up from their leader than others, and I believe we all can need reminders. People are put together in different ways in how we like to get involved in new things that is a bit outside our primary work. For me, it is not very important to have a leader that pats me on the shoulder and makes sure I am doing it (P5).*
More flexible work schedule	*During my workday I am the one to prioritise my time, based on professional considerations of course, and waiting lists and the amount of work. Sometimes, you have the opportunity to perform more detailed assessments in one patient, but most times you can only manage to perform the standard procedure (P10).*
Integration into existing routines	*We have whiteboard meetings twice a week where we go through what we have done and what remains to do (functional assessments, among others). I think that KROSS and vision should be included as an additional whiteboard item …*.* If we see it here, and the assessment is allocated, yes. I believe this can work. And much will be done if vision and KROSS is put it into the routine (P11).*
Further follow-up and supervision in own practice	*It is always challenging to start doing something new. For all of us. And, often that is about feeling confident, at least for me......As such, you need time to practice, and access to resource persons you can contact to supervise and answer questions (P1).*

### Individual barriers

Several individual barriers were expressed by the participants; these barriers were related to competence in different ways: their individual knowledge and skills about visual function and impairments, how they worked with patients with a variety of medical conditions and needs and their previous experiences of other implementation efforts (see Table 2).

The participants expressed that they had a *low competence about visual functions*. This was related to both normal visual function and visual problems in general, particularly VIs after stroke. Regardless of their professional background, they said that vision and visual functions had both had little focus in their education and in later professional work. Several participants reflected that while working with stroke survivors, they wished they possessed better competence in assessing vision to be able to identify whether a problem was related to vision, cognition, communication or physical problems; they expressed this as particularly important when planning rehabilitation for their patients.

Although the participants said they lacked knowledge of visual functions and VIs, some described performing crude assessments of visual function, and others described that they identified vision loss during other practical observations. For example, some physiotherapists observed vision during physical training, and some nurses and assistant nurses observed visual function during meals and activities of daily living (ADL). However, if they suspected VIs, they expressed little confidence in their own observations or test results, and they lacked the language to describe them precisely in the patient’s medical record. The most commonly reported assessment was the waving test (confrontation test) to assess the peripheral visual field. However, this was ‘self-taught’, and they explained they did not fully understand or trust the results because they had a *lack of skills and experience in testing visual functions.*

The participants described themselves as *generalists, not stroke specialists*; this is in contrast to stroke units in hospitals where HCPs can fully admit to stroke care*.* As HCPs in municipal health care services, they had to have general knowledge of many conditions rather than specialist knowledge in one specific field. They expressed a general concern related to their ability to stay professionally updated and provide good enough care because their patients represent a variety of diagnoses with different needs for rehabilitation. They felt a need for more competence in many areas, including stroke, because their patients are discharged from the hospital earlier and are in need of more complex and comprehensive care than just a couple of years ago.

Many participants had some *experience of unsuccessful implementation* projects, and this made them cautious about new implementations. Even in cases where there was an expressed consensus between service leaders and clinicians and where the HCP had signed off that they had read and understood a new routine, it was difficult to maintain sustainable changes. The HCP believed a change of practice was more time-consuming in municipal health care services compared with hospital services and that municipal health care services do not have the same focus on updating their practice. They suggested several explanations, including a lack of formal health education among many staff members, many HCP working part time and a culture resistant to change.

### Individual facilitators

Through a presentation of the project and its aim in meetings and written information about the current study, the participants had been provided with new knowledge about VIs after stroke. When the participants learned about the importance and significance of vision for everyday activities and the consequence and prevalence of VIs after stroke, many highlighted this as a strong motivation for changing their practice. They now said they considered it important to include vision assessment in their practice (see Table 3).

The participants expressed *strong beliefs that including vision in stroke care will provide a better health service* for their patients. When they learned about the significance of VIs after stroke, they expressed it should be an obligation for HCP to change their practice and include a vision assessment in their routines. They also thought that a visual assessment should be done as soon as possible after the stroke because of the implication vision has on other functions, such as mobility, balance and ability to read. All the participants highlighted that knowledge about visual function in and of itself is important when assessing other functions such as balance, language and cognition and when assisting in ADL activities. The participants thought most patients would be positive to have their vision assessed and followed up on because they often wished to return to the life they knew before the stroke and are motivated to do the work required to achieve this. The participants also expressed the importance of including time to build trust between HCP and the patient before performing a vision assessment because many patients are vulnerable after stroke, and they should not be exposed to unnecessary assessments and observations.

One facilitator considered by the participants to be important was if the new vision routine led to positive changes for the patients and further follow-up. *Experiencing new routines to make a difference* must be considered so that the implementation will be worth the invested time and energy to maintain a sustainable routine.

The participants described a need for *experiencing the tool as useful and evidence based.* Some expressed that a new tool and procedure should be based on evidence and were concerned about using a tool not validated for this specific context. Others said it was just as important that the tool felt useful. If experiencing that the patients could benefit from the assessment and results, this would be enough for continuing to use it. Getting access to a visual assessment tool with standardised tests was something the participant’s emphasised as positive, even if the tool was not validated fully. The participants looked forward to performing a more structured and standardised visual assessment than the tests and observations they had previously performed; they said it would improve their knowledge of vision functions and competence of their assessments, bringing confidence to their own observations and assessments. They also commented that a result from a standard test tool would also be easier to communicate to other health care professionals because it could provide a language with known terms to describe vision functions and VIs that they previously did not have.

### Contextual barriers

The contextual barriers represent diverse challenges for implementation that are related to settings outside the individual. The barriers included unclear responsibility of vision care, a need for better interdisciplinary collaboration and formal routines, time constraints and difficulties with the medical record. The analysis showed that the barriers were perceived with different strengths between the three different municipal health care services (see Table 4).

The participants expressed that there was an *unclear responsibility for vision care* and that it was random whether visual function or VIs were being described in hospital transfer records. If vision was mentioned at all, it was often limited to whether the patient needed glasses or not, or when the patient had large visual field defects or neglect. Vision and the assessment of visual function was something the participants initially (before learning about this project) considered to be someone else’s responsibility, for example, the responsibility of the patient’s ophthalmologist or optometrist. Now, they recognised vision as a responsibility for all involved services and professions. Their opinion was also that the hospital stroke units should be responsible for the first vision assessment after stroke. However, they acknowledged that for some, the initial assessment needed to be postponed and performed by the municipal health care service because not all patients are suitable for visual assessments during their hospital stay. They considered this to be a problem because there was currently *a lack of formal stroke routines* in the municipalities. Some stated they had tests and assessments they usually performed and considered important after stroke, but there was no formal stroke patient pathway or guidelines. The participants also experienced that visual function, VIs or recommendations regarding vision rehabilitation were hardly ever described in the patients’ medical record. The participants suggested that along with a formal routine and guideline after stroke, identification and a follow-up of VIs should be included.

Another contextual barrier was the concern that the implementation of visual assessment routines would end up as a task and responsibility for one specific profession and would *lack structured interdisciplinary collaboration*. They considered interdisciplinary collaboration as essential to implement and for securing a visual assessment for all patients because being dependent on one profession could hinder all patients from being assessed. Further, many highlighted the importance of vision assessment being an interdisciplinary matter to raise the awareness, attention and competence of VIs after stroke in the municipality. The participants pointed out the lack of vision specialists within the municipal interdisciplinary team, expressing a need for formal collaboration with vision experts, such as optometrists, vision rehabilitation specialists and ophthalmologists.

All the participants experienced time pressure in their daily routines when it came to caring for an increasing number of patients with complex needs; the fact that the resources in the municipality were scarce only amplified this problem. The participants described different experiences of *time constraints* in their work and their opportunity to add new routines to their practice. For example, nurses and physiotherapists are organised differently in the municipality health care services. The nurses worked shifts every third weekend, while physiotherapists worked regular hours on weekdays. It was also apparent that there was a difference between home care and rehabilitation services. Home care HCP reported having little influence over their own workload and ability to prioritise their tasks. They explained that additional time had to be allocated by their leader if new tasks should be introduced. The time used for an assessment is a factor all the participants agreed had an impact on implementation success, and it should not be too time-consuming. However, what the participants described as an acceptable use of time varied from 15 to 30 min. The ideal time was as short as possible without compromising the quality of the assessment.

A practical barrier was that it was *difficult to integrate the vision tool in the medical record* they used. Results from the KROSS vision assessment should be filled out directly in the form while assessing the patient at the bedside or at home. HCP working in home care already used a tablet to document their work in the patient’s own home; however, it was not possible to include extra assessment forms directly on the tablet. The assessment form would have to be on paper, which they would need to scan or fill in manually to the medical record using the office computer when they got back from the home visit. They considered this a major barrier because it would lead to double work and take away valuable time; more importantly, paper records might be misplaced or lost. In addition, scanned documents are harder to find later when reading the patients’ medical record because of poor digital search abilities in the current municipal medical record system.

### Contextual facilitators

Contextual facilitators were described as different ways of leader support and how some experienced a flexible and autonomous workday. In addition, they suggested integrating the new routine into other routines, such as white board meetings and local competence initiatives (see Table 5). Within these categories there were contextual differences between the services.

The participants expressed good *leader support and acknowledgement* as a facilitator when implementing new routines, but the need for leader involvement differed. Some claimed that an active leader who followed up on the implementation and ensured that everyone adhered to the new routines was crucial. Others emphasised that it was important for them to have the support, trust and understanding from their leader in how they prioritised and spent their time at work, without more detailed follow-up or their leader checking their professional work and decisions; they described this as being allowed to work freely and autonomously.

Although the participants from rehabilitation services all described very busy days, they still had a *flexible work schedule* to prioritise their work. They considered this to be important for implementing a new task, and this would make it possible to include vision assessment and follow-ups into their work routine. In contrast, home care HCP described a more fixed workday with less flexibility to schedule their activities and the content of tasks. This led to a prioritisation of routine tasks and visits, and they expressed reservations in implementing more tasks because this would just add to their already busy days.

When learning specifically about VIs after stroke and new vision assessment routines to be implemented, during the interviews and workshops, the participants discussed how this could be *integrated into existing routines*. Rehabilitation services had, and home care planned to, implement white board meetings where the multidisciplinary team would meet to plan and coordinate their work. The participants suggested that a vision assessment should be included in this meeting. The participants from home care also worked with a standardisation of a first meeting with new patients and suggested integrating a vision assessment in this for new stroke survivors.

All agreed on the importance of feeling confident when performing the assessment; they agreed that *further follow-up and supervision in their own practice* was important in addition to theoretical knowledge and a good user manual to use while testing. They said that feeling insecure in the testing situation may lead to a postponed assessment or them choosing not to do it. Several suggestions on how to secure a follow-up were discussed, including individual supervision in their own practice, plural vision meetings during lunch and easy access to ask the project resource individuals. They also believed that it is important to have more than one person from a workplace to take part in the training; their experience was that if only one person had learned something new, it was difficult to later involve other colleagues.

As presented here and illustrated in Fig. 1, several barriers and facilitators were identified through the interviews and workshops (Phase III). In addition, some strategies to overcome barriers were suggested by the participants, some strategies were identified through the literature, and some were suggested by managers and leaders during Phase II.

## Discussion

Assessing barriers and facilitators is an important part of the implementation process and should be considered when choosing implementation strategies [1]. In the current study, we have identified individual and contextual barriers and facilitators to the implementation of structured visual assessment and follow-up in municipal health care. Some barriers and facilitators seem to influence each other, and combined, these can be important for behaviour change [37].

### Capability and motivation

The participants experienced that they had low knowledge about visual function, lacked the skills needed in vision testing and assessment and acknowledged that they had paid little attention to vision. A review has also documented that vision is given little attention in municipal health care services and that vision specialists are not an integrated part of rehabilitation services [41]. Competence and care for VIs in rehabilitation is described as being less integrated and conceptualised than other outcomes after stroke, such as motor function, language and cognitive impairments [24]. The experience of not being competent and confident when performing a procedure is described as an important barrier for knowledge use [36, 42, 43]. Capability has been described as the individual’s capacity to engage in and perform the behaviour, here performing and including visual assessment and follow-up in their practice [34]. Capability is one part of the COM-B model for behaviour change, with opportunity and motivation as other important parts [44]. Capability includes having the necessary knowledge and skills [34], which the participants in the current study expressed they did not have before the workshop. This may have caused vision routines to be more difficult to implement compared with routines related to clinical areas that HCP are more familiar with.

When the participants learned about the prevalence and consequence of VIs after stroke, they expressed a strong motivation for building their capability and a commitment to provide good quality care and that better routines for VIs should be implemented. Beliefs about consequences is a domain from the theoretical domains framework linked to motivation in COM-B [44]. The experience of vision assessment and the later follow-up being of great significance to the patients’ function and everyday life was an important facilitator expressed by the participants. In the initial rehabilitation process, stroke survivors have been reported as having a strong motivation to return to life as it was before the stroke [45]. This was also something that influenced the participants’ motivation to learn and include new procedures in their work. They also felt that vision health was outside their core task and something optometrists and ophthalmologists had the responsibility for. However, now, they had learned that vision was a prerequisite for other rehabilitation efforts, and they stated that it should be a part of municipal rehabilitation and care. One major barrier described by the participants was the unfamiliarity with the vision terminology and lack of language to describe their observations related to VIs. This reduces both the opportunity to change behaviour [44], potentially hindering collaboration and efficient vision rehabilitation. The participants acknowledged that the KROSS tool could be a useful tool, improving their capability to describe their assessment of visual function and VIs.

Evidence considered strong by knowledge users has been shown to be more easily adopted than practice with weak evidence [46]. However, as others have described, evidence from research is not sufficient alone, clinical competence and experiencing that the evidence is useful in practise is important [47]. Our study shows that the participants weighted the experience of usefulness higher than strong evidence as a motivator for implementation. On the other hand, if the new routine is not seen as making a difference and is experienced only as a formality, it will not be considered useful and instead just another “tick off” task [46]. This is new knowledge that elucidates evidence as a motivator for adopting new clinical routines, particularly when strong evidence is lacking.

Our findings suggest a lack of focus and competence regarding vision and VIs in health care services and education. Considering that the visual sense is of such importance for function, quality of life and wellbeing, vision deserves more attention from HCP and educational institutions [7, 8, 13–15, 21, 47].

### Contextual differences within municipal health care services

The HCP in the present project represent three different contexts within the municipal health care organisation: the in-patient rehabilitation unit, home-based rehabilitation and home care. In our findings, the same barriers and facilitators are present and central in all three contexts, but in particular, some contextual barriers and facilitators were more distinct for one context than others. This is important to consider when developing implementation strategies in municipal health care settings.

As in the present project, practical organisational barriers are frequently reported [42, 48–50]. A concrete problem in the current project was including the KROSS tool as a digital file in the medical record. Alternatives to storing the file were discussed with leaders and practitioners, and a procedure was agreed upon during the workshops. However, it became apparent that this procedure would not work for home care because they use a tablet for all documentation during home visits, and currently, there were no technical solutions to add the recording of the vision tool to the tablet. The solution for home care was that they would have to use a paper copy during home care visits and manually add this to the medical record when they came back to their office. In home care, this may be an additional barrier. Failing to integrate the assessment in the medical record makes it hard to find the information again and may hinder the active use of the results from the assessment.

Limited time and resources are well-known contextual barriers for implementation [44]. The participants expressed different opportunities and abilities to prioritise their own time and workday. Especially the participants working in home care reported that their lists were so full that new things were difficult to include. The participants in the rehabilitation service described time constraints differently. Because they were more autonomous in their ability to organise their daily work schedule, they were more flexible in how they could organise their day, although they also experienced time constraints. The experience of limited time and high levels of stress in home care is also documented in other studies [51, 52]. The participants suggested that 15–30 min could be appropriate for a structured vision assessment.

### Leader support

Leader involvement was something the participants viewed as essential for change of practice although they had different opinions on how the leader should be involved. There are many ways a leader could be involved in an implementation project [53]. In Norwegian municipal health care services, managers often have a health care education themselves; however, because their role as leaders is more about organising their department, they tend to delegate responsibility for competence improvement and quality of care to the other HCP in their department [54]. Some participants expressed that it was important to have a leader who was closely involved in their daily work, and they described this as reassuring, particularly during the implementation of new routines. However, others expressed that they preferred to work professionally autonomously with the trust of their leader, rather than having a leader who checked the details of their work. Regarding being involved in implementing the new vision assessment routines in the project, all the participants agreed it was important for them that their leader supported and facilitated their participation in this implementation project. A recent Norwegian study also highlights that an empowering leader is a facilitator for implementation in municipal health care services [55].

### Importance of actively integrate partners to facilitate implementation

In this implementation project all partners were actively involved in all phases of the implementation, in line with the KT process [33]. This provided valuable insight and elucidation of barriers and facilitators that might have been missed without this partnership. Involving HCP allowed us to come up with practical solutions to promote facilitators and overcome barriers. One example was to include the KROSS vision assessment as a fixed point during the daily white board meetings. During the white board meetings, the interdisciplinary staff discuss patients and plan their activities as written on the white board (https://pasientsikkerhetsprogrammet.no/forbedringskunnskap/Tavlemoter). The rehabilitation services had already started to use white board meetings successfully, and the home care services were about to start. Another example was that lunch discussions and local workshop would enhance the knowledge use. This active partnership including HCP are more likely to promote a sustainable change to clinical practise [33, 56].

This implementation has an ambitious and important goal [13, 57] with several aspects. At a very early stage, we established contact and cooperation with different partners, including leaders and practitioners in the municipality and user groups. The involvement of partners provided early and continued dissemination throughout the project of its aim, which allowed the participants to reflect on their practise towards VIs after stroke. In preparation for the current study this provided partner involvement in planning and performing the interviews and workshops. These activities secured that the project was anchored in the organisation in order to promote a successful implementation.

### Strengths and limitations

The current study has a relatively small sample size, and the barriers and facilitators described in the present study are based on the participants’ descriptions of their practice, competence and experience. Their experiences might not be representative of the municipal service as a whole; however, the participants were purposefully recruited, aiming for a broad description of practice and potential barriers and facilitators. The interviews were rich and detailed and represented the different contexts and organisational levels in the municipal health care services. The results confirmed previously known determinants and elucidated others: knowledge and competence of vision and the implications of living with vision impairments after stroke were a strong motivator and facilitator. The results from the interviews were presented and discussed at the workshops, where there was a wider representation of municipal HCP and where the determinants and implementation strategies were recognised and elaborated on. One challenge is that the described determinants may differ from the actual determinants that will come up during the implementation (Fig. 1, Stage IV, paper in preparation). Even though Stage IV may find a difference between the expected and actual determinants, identifying the determinants and designing strategies to remove or reduce barriers and to strengthen facilitators is an important part of successful implementation in health care services [42]. The current study was done in a medium-sized municipal in Norway, and other municipalities and health care organisations may have different contextual challenges. Still, there are reasons to believe that many health care services outside of Norway may have similar challenges because other studies support that vision and visual function are not prioritised in municipal health care [13, 58, 59]. Further, providing a plan for increased competence and an assessment tool can improve vision care from non-vision experts [60]. A strength of this study is the strong and active partner integration with extensive cooperation with different partners within the municipality and stroke survivors. Strong partner involvement is important to consider if the results should be used to implementation projects in other municipalities.

## Conclusion

The current study shows that low knowledge about VIs after stroke and competence in testing visual function are potential barriers to implementing new vision routines in municipal health care services. Increased knowledge about VIs’ significance for stroke survivors, and a strong motivation to provide best possible care and rehabilitation were important individual facilitators. Contextual barriers can be practical and related to limited time and resources. Supportive management and utilising existing systems to include new routines may facilitate knowledge use. Knowledge from this study will be used in the KT process, to help select, tailor and implement structured vision assessment with the KROSS tool. Strong collaboration with partners in all the KT phases were vital to gain insights into relevant barriers and facilitators, and needs to be considered when planning to implement structured visual assessment after stroke in municipal or primary health care services.

## Supplementary Information

**Additional file 1.**

**Additional file 2.**

## Data Availability

The transcripts and notes used and analysed during the current study are not publicly available due to protection of the anonymity of the participants, and the content may threaten confidentiality. An anonymised version of the data can be made available from the corresponding author on reasonable request.

## Abbreviations

HCP: Health care professional
IKT: Integrated knowledge translation
KROSS: Competence, Rehabilitation of Sight after Stroke
KT: Knowledge translation
KTA: Knowledge to action
VIs: Visual impairments
VISA: Vision impairment screening assessment
