# Supplementary material for: Barriers and facilitators to the implementation of a structured visual assessment after stroke in municipal health care services
Source: BMC Health Serv Res. 2021 May 24;21:497. doi: 10.1186/s12913-021-06467-4 (PMC8147019; doi:10.1186/s12913-021-06467-4)
Supplement: Supplementary file 1 — Additional file 1. [file 12913_2021_6467_MOESM1_ESM.docx]

# Barriers and facilitators to the implementation of a structured visual assessment after stroke in municipal health care services

Topic guide individual interviews. We are interested in your experiences of vision care within the municipal health services, as a part of the implementation of a structured visual assessment after stroke. Please take all the time necessary for your answers.

**Section 1: Your role and tasks in stroke care**

How do you experience your role and task in stroke care? Can you describe the stroke care in the municipal health services?

*Optional subquestions*

- Work place, education and experience
- Which part of municipal health care services do you work in?
- Where in the stroke care pathway do you provide your services to the stroke survivor?
- How are stroke survivors referred to your services?
- Can you describe what kind of health services do you provide to the stroke survivors?
- Are you a part of an interdisciplinary team?
  - If so, what is your responsibility within this team?
  - How is the team organised?
  - Which other professions do you work with?
  - Are you involved with working with the patients’ individual rehabilitation plan?

**Section 2: Experiences with the present practise of visual assessments and follow up of visual impairments**

I would like you to consider todays practice. Can you describe the present practise of visual assessments and follow up of visual impairments? What are your experiences with assessment and follow up of vision impairments after stroke? Who would you consider is, or should be, responsible for vision assessments following stroke?

*Optional subquestions*

- If you assess vision, is it done systematically, or when you suspect visual impairments?
- Can you reflect on whether you consider visual assessment important?
- Related to the transfer of patients to you, do you have access to information about the visual function of your patients?
- If you do get information, can you describe who provides it (e.g. the hospital, service allocation office, general practitioner)?
- If you perform any visual assessments today, can you describe this in more detail?
- Do you use any vision assessment tools at present, if so please describe?
- If you are part of an interdisciplinary team, is visual function something you assess, or is this done by others (specify)?

**Section 3: Experiences with assessment tools**

If you use assessment tools, can you describe your experience of using them in your practise today? What tools do you use, and why do you use them?

*Optional subquestions*

- If you do not have an assessment tool for vision, do you have any suggestions to how an assessment tool for assessing visual function after stroke should be?
- Can you describe how you document results from standardised assessment tool?
- What are your thoughts on including a new vision assessment tool to your practise?
- What may, or may not, contribute to use of the tool? Can you give examples?

**Section 4: Experiences with competence improvement and implementation**

Thinking back to other competence improvements and implementation projects in your service. Are there things important to consider when implementing new vision routines?

*Optional subquestions*

- What do you consider important for including vision assessment and follow up for stroke survivors in your practise?
- Can you describe your experiences with earlier competence improvement or implementation projects in your practise?
- Can you tell if anything have influenced negatively on earlier implementation projects?
- What do you believe is important for this vision implementation project to succeed (Number of participants, follow up from project leaders….)?
- How can practitioners train and supervise their co-workers for sustaining the new practise?

**Closure:** Anything you wish to include that we have not discussed during the interview.

**Workshop discussion**

After being presented with preliminary results from the interviews the participants were asked for their views in a plenary discussion, with particular focus on strategies and suggestions for a successful implementation.

Implementation outcome: All stroke survivors should have their vision assessed and followed up if needed.

*Optional subquestions*

- When is the proper timing for the assessment?
- Who should perform the assessment?
- How can we secure proper follow up?
- How can you store the assessment in the electronic journal?
- Potential barriers for implementation
- Potential facilitators for implementation
