# Supplementary material for: Barriers and facilitators to the implementation of a structured visual assessment after stroke in municipal health care services
Source: BMC Health Serv Res. 2021 May 24;21:497. doi: 10.1186/s12913-021-06467-4 (PMC8147019; doi:10.1186/s12913-021-06467-4)
Supplement: Supplementary file 2 — Additional file 2. [file 12913_2021_6467_MOESM2_ESM.pptx]

## Slide 1
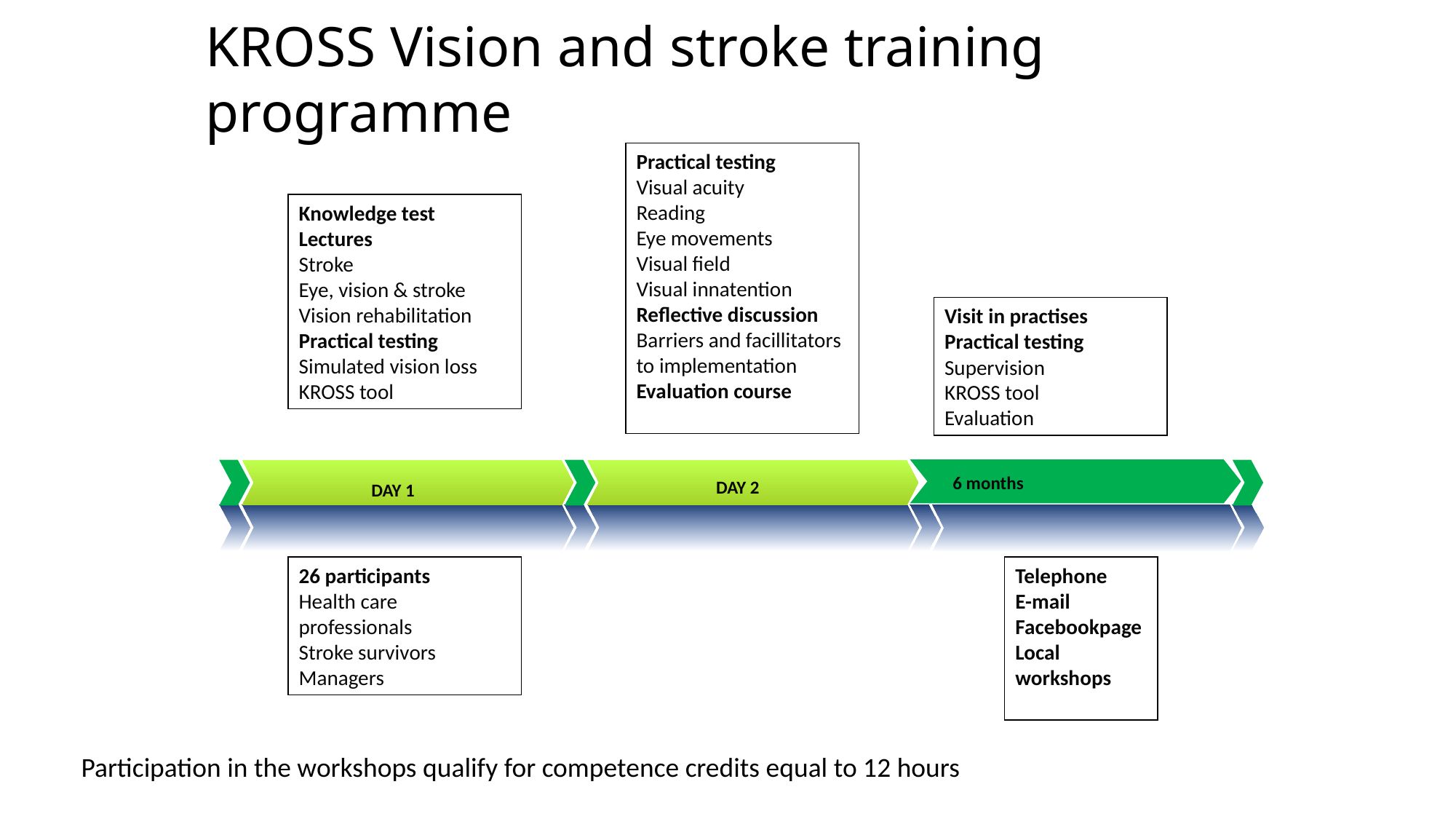

KROSS Vision and stroke training programme
Practical testing
Visual acuity
Reading
Eye movements
Visual field
Visual innatention
Reflective discussion
Barriers and facillitators to implementation
Evaluation course
Knowledge test
Lectures
Stroke
Eye, vision & stroke
Vision rehabilitation
Practical testing
Simulated vision loss
KROSS tool
Visit in practises
Practical testing
Supervision
KROSS tool
Evaluation
DAY 2
DAY 1
6 months
26 participants
Health care professionals
Stroke survivors
Managers
Telephone
E-mail
Facebookpage
Local workshops
Participation in the workshops qualify for competence credits equal to 12 hours
